# The Relationship between Grandiose and Vulnerable (Hypersensitive) Narcissism

**DOI:** 10.3389/fpsyg.2017.01600

**Published:** 2017-09-13

**Authors:** Emanuel Jauk, Elena Weigle, Konrad Lehmann, Mathias Benedek, Aljoscha C. Neubauer

**Affiliations:** Department of Psychology, University of Graz Graz, Austria

**Keywords:** grandiose narcissism, vulnerable narcissism, hypersensitive narcissism, extraversion, introversion

## Abstract

Narcissistic grandiosity is characterized by overt expressions of feelings of superiority and entitlement, while narcissistic vulnerability reflects hypersensitivity and introversive self-absorbedness. Clinical evidence suggests that grandiosity is accompanied by vulnerable aspects, pointing to a common foundation. Subclinical personality research, however, views grandiose and vulnerable narcissism as independent traits. Grandiose narcissism displays substantial correlation with extraversion, while vulnerable narcissism correlates highly with introversion. We investigated if (1) controlling for intro-/extraversion might reveal a “common core” of grandiose and vulnerable narcissism, and if (2) the correlation between both aspects might be higher at higher levels of narcissism. Latent variable structural equation modeling and segmented regression analysis confirmed these hypotheses in a large non-clinical sample (*N* = 1,006). Interindividual differences in intro-/extraversion mask the common core of grandiose and vulnerable narcissism. The association between both aspects increases at high levels (upper 10%) of grandiose narcissism, which suggests a possible transition to clinically relevant (pathological) narcissism.

## Introduction

When hearing the word “narcissism,” most people think of exaggerated self-worth, feelings of grandiosity and superiority, admiration seeking, as well as entitlement and arrogance (cf. Buss and Chiodo, 1991; Pincus et al., 2014), which circumscribes the subclinical concept of *grandiose narcissism* (or overt narcissism; Raskin and Hall, 1981; Wink, 1991). Besides grandiose narcissism, there is the less prominent concept of *vulnerable narcissism* (also termed hypersensitive or covert narcissism; Wink, 1991; Hendin and Cheek, 1997). Vulnerable narcissism also entails pronounced self-absorbedness, but apart from that, goes along with self-consciousness, social insecurity, and defensiveness. Though both constructs share the central concept of self-centeredness (e.g., Brown et al., 2016), they phenomenologically manifest in very different personality types^1^, which Wink (1991) originally referred to as the “two faces” (p. 590) of narcissism: subclinical grandiose narcissists are extraverted, socially bold, and even charming (e.g., Back et al., 2010; Dufner et al., 2013; Jauk et al., 2016b). Vulnerable narcissists, on the contrary, are introverted, defensive, and avoidant (Miller et al., 2011, 2012; Hart et al., 2017). While grandiose narcissism displays substantial and consistent correlations with extraversion (Raskin and Hall, 1979, 1981; Emmons, 1984; Paulhus, 2001; Paulhus and Williams, 2002; Schütz et al., 2004; Lee and Ashton, 2005; Ackerman et al., 2011; Miller et al., 2011; Roche et al., 2013; Zajenowski et al., 2016), vulnerable (hypersensitive) narcissism is consistently associated with introversion (Wink, 1991; Hendin and Cheek, 1997; Given-Wilson et al., 2011; Miller et al., 2011; Zajenowski et al., 2016). Population-based studies using non-clinical samples further show that grandiose and vulnerable narcissism are essentially unrelated (Hendin and Cheek, 1997; Miller et al., 2011; Ng et al., 2011; Brown et al., 2016; Ksinan and Vazsonyi, 2016), why they are commonly viewed as two independent traits in subclinical personality research.

In contrast, clinical evidence suggests that grandiose narcissism is always accompanied by vulnerable aspects (Kernberg, 1975; Pincus and Lukowitsky, 2010; Roberts and Huprich, 2012; Pincus et al., 2014; Gore and Widiger, 2016). Specifically, clinical observations show that patients diagnosed with narcissistic personality disorder (NPD), a pathological form of narcissism, display co-occurring or oscillating states of grandiosity and vulnerability (Pincus and Lukowitsky, 2010). Recent systematical investigations confirm that this is particularly true for individuals identified as grandiose narcissists, who display episodes of vulnerability (Gore and Widiger, 2016). Accordingly, scales designed to assess pathological narcissism, such as the Pathological Narcissism Inventory (PNI), encompass vulnerable aspects such as *contingent self-esteem* or *hiding the self* alongside grandiose aspects such as *self-sacrificing self-enhancement* or *grandiose fantasy* (Pincus et al., 2009). These scales show distinct correlations with measures of grandiose and vulnerable narcissism designed for population-based studies, most notably the Narcissistic Personality Inventory (NPI; Raskin and Hall, 1981) and the Hypersensitive Narcissism Scale (HSNS; Hendin and Cheek, 1997). However, to date, little is known about the transition from normal to pathological narcissism (Pincus and Lukowitsky, 2010).

If narcissistic grandiosity is, as clinical evidence suggests, always accompanied by vulnerability, this should manifest in a correlation between measures of grandiose and vulnerable narcissism, but this is not the case in subclinical samples (e.g., Miller et al., 2011). In clinical samples diagnosed with NPD, the correlation between grandiose and vulnerable narcissism has not yet been investigated systematically [quantitative studies reporting correlations between grandiose and vulnerable narcissism in clinical samples typically encompass patients with any mental disorder rather than NPD (e.g., Fossati et al., 2009)]. Given the evidence reviewed above, we hypothesize that there might be two causes for the lack of correlation between grandiose and vulnerable narcissism in the general population: (1) The correlation between grandiose and vulnerable narcissism might be masked by interindividual differences in intro-/extraversion – a variable that has consistently been shown to correlate with both constructs. (2) The association might be non-linear across the narcissism distribution, with no correlation at the lower end, and an increasing correlation toward the upper end. We elaborate on these hypotheses in the following.

First, we propose that the correlation between grandiose and vulnerable (hypersensitive) narcissism is masked by interindividual differences in intro-/extraversion in the general population, which is substantiated by different theoretical and empirical considerations: (a) Theoretical accounts of grandiose and vulnerable narcissism both emphasize self-centeredness as a core feature (Miller et al., 2014b; Krizan and Herlache, 2017), but this common core is not evident in subclinical personality research. (b) Grandiose and vulnerable narcissism both display pronounced correlations with extra- and introversion, respectively, but not with each other. (c) Intro-/extraversion and narcissism differ with respect to their foundations as personality traits: intro-/extraversion is a basal and broad trait that is highly genetically determined (McCartney et al., 1990; Loehlin, 1992), biologically rooted (Eysenck, 1967), and manifests in early infancy (Kagan, 1994). Narcissism, on the contrary, is a narrower trait and is assumed to be determined socially, primarily due to parenting style (Kernberg, 1975; Kohut, 1977; Otway and Vignoles, 2006; Brummelmann et al., 2015a,b) and further cultural socialization factors (Twenge and Campbell, 2009; Twenge and Foster, 2010). From a developmental perspective, intro-/extraversion thus can be expected to develop ontogenetically earlier than narcissism (which emerges in late childhood; Thomaes et al., 2013). As a consequence, intro- and extraverted individuals may respond differently to social influences that lead to core narcissistic beliefs (“I am superior”) and develop either an extraverted narcissistic style (grandiose narcissism) or an introverted narcissistic style (vulnerable narcissism). Based on a large cross-sectional sample, we investigate this hypothesis by means of latent variable structural equation models (SEMs) in which we adjust the latent correlation between grandiose and vulnerable narcissism for interindividual differences in intro-/extraversion.

To investigate the discriminant validity (specificity) of this hypothesis, we repeat the analyses for the personality dimension of neuroticism. Neuroticism is also frequently reported to correlate negatively with grandiose, but positively with vulnerable narcissism (e.g., Miller et al., 2011, 2015; see also Gore and Widiger, 2016). It could thus be hypothesized that controlling for neuroticism might increase the correlation between grandiose and vulnerable narcissism as well, as these could be regarded as emotionally stable and unstable forms of narcissism. However, we do not expect this effect to be particularly strong, as grandiose and vulnerable (hypersensitive) narcissism, are, for the reasons provided above, possibly better understood in terms of other-directed (extraverted) versus self-directed (introverted) narcissism. We thus expect that controlling for interindividual differences in neuroticism will not alter the correlation between grandiose and vulnerable narcissism substantially.

Second, we investigate whether the association between grandiose and vulnerable narcissism differs as a function of the level of narcissism: we hypothesize that the correlation is higher toward the upper end of the grandiose narcissism distribution. Besides clinical evidence suggesting that the two constructs covary particularly when it comes to pathological narcissism (i.e., high levels of narcissism), a recent systematic investigation found that individuals identified as grandiose narcissists are likely to display episodes of vulnerable narcissism as well (cf. Gore and Widiger, 2016). We use segmented regression analysis, a statistical technique that allows for an empirical detection of a breakpoint (i.e., a change in slope) in a bivariate regression relationship, to test for a change in slope in the bivariate distribution of grandiose and vulnerable narcissism. Importantly, we perform this analysis in a large (*N* > 1000) and non-clinical sample that covers a broad range of the narcissism distribution. While the majority of this sample displays, per definition, normal (i.e., non-clinical) personality variation of narcissism, the very top can be assumed to display potentially clinically relevant narcissism scores^2^. Continuous variation is an important prerequisite for segmented regression analysis and allows for a powerful test of a possible change in slope in linear associations (cf. Jauk et al., 2013).

## Materials and Methods

### Participants and Procedure

Participants took part in one of four studies (two offline, two online; see **Table 1**) at the University of Graz. We combined data from multiple studies to enable a powerful test of latent relationships and of slope differences across the whole range of narcissism. The online studies were performed using Limesurvey, data in the offline studies were collected by students at the University of Graz using standardized test booklets. This study was carried out in accordance with the relevant guidelines and recommendations with written informed consent from all subjects. All subjects gave written informed consent in accordance with the Declaration of Helsinki. The protocol was approved by the ethics committee of the University of Graz. Each of the studies had different research aims, but all entailed the same measures of grandiose narcissism and vulnerable narcissism. The total sample included *N* = 1,006 (550 women) participants with a mean age of 24.06 (*SD* = 5.03) years. Studies one, two, and three additionally encompassed a measure of intro-/extraversion (*N* = 605 [350 women], mean age of 23.53 [*SD* = 4.33] years); studies one and two also entailed a measure of neuroticism that we used for complemental discriminant validity analyses (*N* = 406 [202 women], mean age of 23.58 [*SD* = 3.28] years). The percentage of missing data was low (not exceeding 1.5% for any single item). **Table 1** shows detailed sample characteristics. We observed mean differences in the variables under study across the four samples (most notably between the online and offline samples). However, our research question concerns the covariance structure of the data, which was found to be invariant across studies (see Results).

**Table 1:** Descriptive statistics across the four studies and intercorrelations of the study variables.

	Study I (*n* = 204, offline)		Study II (*n* = 202, offline)		Study III (*n* = 199, online)		Study IV (*n* = 401, online)							
											
	*M*	*SD*	*M*	*SD*	*M*	*SD*	*M*	*SD*	*p*	Post-test	2	3	4	5	6
Sex (1)	1.50	0.50	1.51	0.50	1.26	0.44	1.50	0.50	<0.001	1,3; 2,3; 3,4	**0.14**	**0.24**	-0.06	0.02	**-0.54**
Age (2)	24.26	3.56	22.88	2.82	23.43	5.92	24.85	5.85	<0.001	1,2; 2,4; 3,4		0.06	**-0.08**	**0.12**	**-0.22**
Grandiose Narcissism (3)	14.92	6.58	15.55	6.78	13.22	6.58	16.13	6.80	<0.001	2,3; 3,4			-0.03	**0.47**	**-0.33**
Vulnerable Narcissism (4)	2.65	0.54	2.74	0.57	2.86	0.59	2.76	0.55	0.001	1,3				**-0.48**	**0.30**
Intro-/Extraversion (5)	3.62	0.60	3.62	0.59	3.38	0.72	–	–	<0.001	1,3; 2,3					**-0.14**
Neuroticism (6)	3.07	0.72	3.08	0.73	–	–	–	–	0.870	–					


*Posttest-column denotes significant differences between studies by means of Bonferroni-corrected post-tests. Correlations are based on *N* = 1,006 except for correlations encompassing intro-/extraversion (*N* = 605 without Study IV) and neuroticism (*N* = 406 for studies I and II). Sex was coded 1 for women, 2 form men. High values on Intro-/Extraversion denote extraversion. Significant correlations at *p* < 0.05 are printed in bold face.*

### Measures

Grandiose narcissism was assessed with the German Narcissistic Personality Inventory (NPI; Schütz et al., 2004). The NPI comprises 40 dichotomous forced-choice statements. The internal consistency of the scale was α = 0.84 at manifest level. Vulnerable narcissism was assessed with a German translation of the Hypersensitive Narcissism Scale (HSNS; Hendin and Cheek, 1997). The reliability of the 10-item scale was α = 0.66 for the manifest scale (like in the original publication; 0.63 < α < 0.75; Hendin and Cheek, 1997). The NPI and the HSNS can be considered the long-time standard measures of grandiose and vulnerable narcissism in non-clinical personality research and were found to have very good concurrent validity with expert ratings of narcissism (Miller et al., 2014a). Intro-/extraversion and neuroticism (emotionality) were assessed using the 10-item scales of the German HEXACO Personality Inventory (HEXACO-60; Ashton and Lee, 2009); the internal consistencies were α = 0.81 for intro-/extraversion and α = 0.73 for neuroticism at manifest level.

### Data-Analytical Strategy

#### Structural Equations Models

We sought to investigate the correlation between grandiose and vulnerable narcissism with and without consideration of intro-/extraversion by means of latent variable structural equation modeling. For this, we examined two equivalent (i.e., same fit indices) SEMs: one with intro-/extraversion as a correlate of grandiose and vulnerable narcissism, and one with extraversion as a predictor of both variables. Prediction, in this model, is not meant to reflect causality, but allows controlling for interindividual differences in extraversion: as soon as extraversion is defined as a predictor variable, variance in grandiose, and vulnerable narcissism is parted into explained and unexplained variance (i.e., residual variance). We specified a correlation between the two residuals terms, i.e., the variance in grandiose and vulnerable narcissism that cannot be accounted for by differences in intro-/extraversion. By these means, it is possible to estimate the latent correlation between grandiose and vulnerable narcissism while controlling for interindividual differences in intro-/extraversion (paralleling a partial correlation analysis at manifest level; cf. Benedek et al., 2014).

The same analyses were repeated for the personality dimension of neuroticism to investigate the discriminant validity (specificity) of our hypothesis. The SEMs exactly correspond to those reported above, but with neuroticism (instead of intro-/extraversion) as a personality predictor. We assumed that controlling for neuroticism would not alter the correlation between grandiose and vulnerable narcissism substantially.

Finally, in a last step, we specified another SEM with intro-/extraversion and neuroticism as simultaneous predictors of grandiose and vulnerable narcissism, thus controlling for the shared variance of the predictor variables. This model helps to understand the relative contributions of both variables in a more comprehensive manner.

Latent variable modeling was performed in Mplus 7 using maximum likelihood estimation with robust standard errors (MLR) to account for skewness in the manifest variable distributions. We followed a two-step modeling procedure in which identified parts of the measurement models were evaluated first (Anderson and Gerbing, 1988). We assessed model fit using the χ^2^ test, the comparative fit index (CFI), the root mean square error of approximation (RMSEA), and the standardized root mean square residual (SRMR) (Hu and Bentler, 1998, 1999; Beauducel and Wittmann, 2005).

**FIGURE 1:**
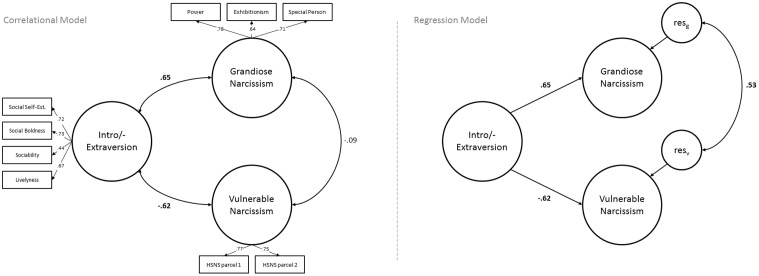
Structural equation models (SEMs) displaying the latent correlations between grandiose and vulnerable narcissism with extraversion as a covariate (correlational model, **left**) and as a control variable (regression model, **right**). Measurement models are not displayed in the regression model as they do not differ from the correlational model. HSNS, Hypersensitive Narcissism Scale; res_g_, residuum grandiose narcissism; res_v_, residuum vulnerable narcissism.

**Figure 1** shows the specification of the measurement models. Intro-/extraversion was modeled at facet level on the basis of the factor structure of the HEXACO-60 (2–3 items per facet; see Ashton and Lee, 2009); four error correlations^3^ were allowed to deviate from zero. Grandiose narcissism was also modeled at facet level using the three-factor structure of the NPI proposed by Kubarych et al. (2004; see also Corry et al., 2008). Vulnerable narcissism was modeled using two item parcels (odd and even items).

We tested for measurement invariance of the covariance structure across the three samples (studies one, two, and three) using multigroup modeling. All factor loadings, error correlations, and correlation/regression coefficients between the latent variables were first estimated freely and then constrained to equality across groups. If the model fit of the constrained model does not differ significantly from that of the unconstrained model, we can assume measurement equivalence across samples, supporting the pooling of data across studies.

#### Segmented Regression Model

To test a possible non-linear relationship between grandiose and vulnerable narcissism, we applied segmented regression analysis. Segmented regression is an iterative computational procedure that allows for the detection of a breakpoint, i.e., a significant change in slope, in a continuous bivariate distribution (Ulm, 1991; Haybach and Küchenhoff, 1997). “Segmented” hereby refers to the assumption that a given regression function *Y* = *f(X)* has different parameters in different segments of the independent variable *X*. It is commonly used in epidemiology, for instance, to investigate dose-response research questions such as *at which breakpoint* ψ *does a stressor X have an impact on health outcome Y?* Segmented regression was previously used in psychology to investigate the non-linear relationship between intelligence and creativity, for instance (i.e., how much intelligence does it take to be creative?; Jauk et al., 2013; Karwowski et al., 2016). In the present data, we hypothesized that the correlation between grandiose and vulnerable narcissism would increase toward the upper end of the NPI distribution, because clinical evidence suggests that excessive grandiose narcissism is accompanied by vulnerable aspects (Pincus and Lukowitsky, 2010), but not necessarily the other way around (Gore and Widiger, 2016).

The breakpoint model was estimated using the *segmented* package (Mueggo, 2008) for the open statistics software R (RStudio Version 0.99.446). Grandiose narcissism served as the independent, vulnerable narcissism as the dependent variable. The algorithm has to be supplied with an (arbitrary) initial guess parameter for the breakpoint. We used an initial guess parameter of ψ_0_ = 15 points in the NPI raw score^4^. The empirically determined breakpoint was tested for statistical significance by means of the Davies test (Davies, 1987). This test estimates the probability of a significant change in slope (*H*_1_) under the assumption that the breakpoint parameter ψ vanishes under *H*_0_. The Davies test has to be supplied with a number of *K* equally spaced evaluation points between the 5 and 95% quantiles of the independent variable. According to common recommendations this parameter was set to *K* = 7 (Mueggo, 2008). Significance tests were performed two-tailed at α = 0.05. Because the analysis can be sensitive to influential data points, we checked for outliers in the bivariate distribution by means of Mahalanobis Distance (cf. Jauk et al., 2013). One individual (scoring high on both the NPI and the HSNS) exceeded Mahalanobis distance at *p* < 0.001 and was thus excluded from further analyses. Further precursor analyses showed that the use of segmented regression is justified as the correlation between the NPI and the HSNS can be better explained by adding a quadratic above the linear term (Δ*R*^2^ = 0.01, *p* = 0.002; cf. Jauk et al., 2013). Data and syntax to this study can be obtained via the open science framework osf.io/t474b.

## Results

### Structural Equation Models

#### Intro-/Extraversion

The unconstrained multigroup SEM (different coefficients across studies) converged to an admissible solution and showed acceptable fit to the data [χ^2^(72) = 187.73, *p* < 0.001; CFI = 0.93; RMSEA = 0.07; SRMR = 0.07]. Although the χ^2^ test was significant, fit indices are of similar magnitude to previous studies investigating personality constructs, especially the NPI (Corry et al., 2008). The constrained model (coefficients constrained to equality across studies) displayed similar fit to the data [χ^2^(102) = 223.60, *p* < 0.001; CFI = 0.92; RMSEA = 0.06; SRMR = 0.11] and did not fit the data significantly worse according to the Satorra–Bentler – scaled difference test for MLR estimation [Δχ^2^(30) = 39.39, *p* = 0.12]. Thus, measurement invariance between the covariance structures of the different samples can be assumed, and all further analyses are reported for the whole sample.

**Figure 1** depicts two SEMs (non-multigroup SEM, to obtain single coefficient estimates) with extraversion as a covariate (correlational model) and as a predictor variable (regression model). All factor loadings are significant at *p* < 0.001. The latent correlations between intro-/extraversion and grandiose (*r* = 0.65, *p* < 0.001) as well as vulnerable (*r* = -0.62, *p* < 0.001) narcissism are of similar magnitude, but in opposing directions. The latent correlation of *r* = -0.09 between grandiose and vulnerable narcissism is insignificant in the correlational model (*p* = 0.22). This correlation rises to *r* = 0.53 (*p* < 0.001) as soon as differences in intro-/extraversion are controlled in the regression model.

#### Neuroticism

Again, we first tested for invariance of the structural models across studies. The fit of the constrained model [χ^2^(66) = 192.81, *p* < 0.001; CFI = 0.83; RMSEA = 0.10; SRMR = 0.09] did not deviate from that of the unconstrained model [χ^2^(56) = 178.38, *p* < 0.001; CFI = 0.84; RMSEA = 0.10; SRMR = 0.08; Δχ^2^(10) = 15.46, *p* = 0.11], why measurement invariance can be assumed. Model fit can be deemed acceptable for the sake of this complemental analysis.

Again, in the correlational model (not displayed; all factor loadings significant at *p* < 0.001), the latent correlations between neuroticism and grandiose (*r* = -0.45, *p* < 0.001) as well as vulnerable (*r* = 0.37, *p* < 0.001) narcissism were of similar magnitude, but in opposing directions. This model yielded an insignificant latent correlation of *r* = 0.01 (*p* = 0.91) between grandiose and vulnerable narcissism. In the regression model, this correlation increased to a significant correlation of *r* = 0.21 (*p* = 0.01).

#### Intro-/Extraversion and Neuroticism

**FIGURE 2:**
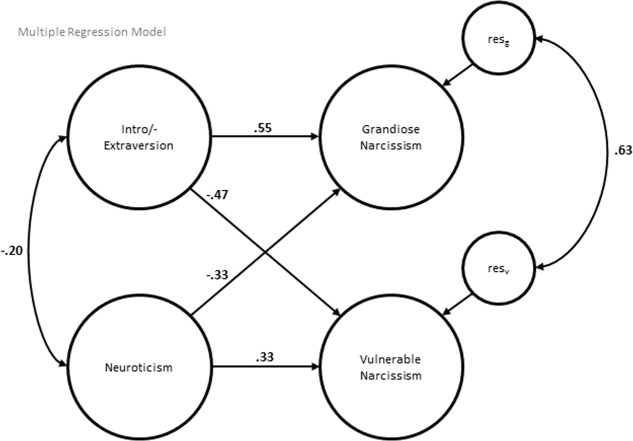
Latent multiple regression model for examining the relative effects of intro-/extraversion and neuroticism. Measurement models are not displayed. res_g_, residuum grandiose narcissism; res_v_, residuum vulnerable narcissism.

Finally, we also set up a model with intro-/extraversion and neuroticism as simultaneous predictors of grandiose and vulnerable narcissism [unconstrained: χ^2^(119) = 339.51, *p* < 0.001; CFI = 0.83; RMSEA = 0.10; SRMR = 0.08; constrained: χ^2^(140) = 351.53, *p* < 0.001; CFI = 0.84; RMSEA = 0.09; SRMR = 0.09, Δχ^2^(21) = 18.12, *p* = 0.64]. Again, model fit was not as good as in the main analysis, but can be considered acceptable. As **Figure 2** shows, there was a negative correlation between intro-/extraversion and neuroticism. The effects of intro-/extraversion on grandiose and vulnerable narcissism were generally stronger than those of neuroticism. The correlation between grandiose and vulnerable narcissism increased to *r* = 0.63 (*p* < 0.001) after controlling for both personality traits.

### Segmented Regression Model

**FIGURE 3:**
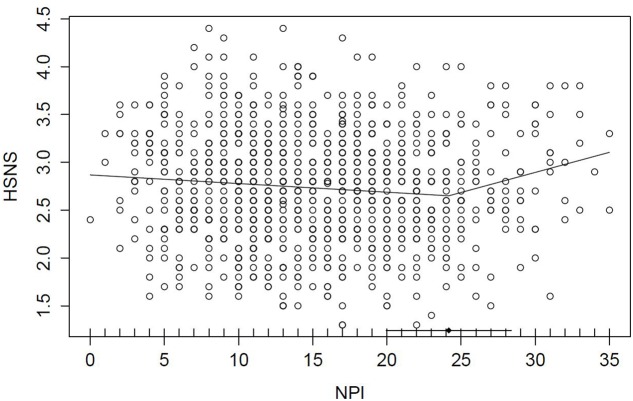
Segmented regression model denoting the breakpoint in the association between the Narcissistic Personality Inventory (NPI) and the Hypersensitive Narcissism Scale (HSNS). Horizontal lines indicate 95% CI of the breakpoint.

To investigate a possible non-linear relationship between grandiose and vulnerable narcissism, we tested for a breakpoint in the continuous bivariate distribution using segmented regression analysis. The breakpoint was estimated at an NPI raw score of 24.17 points (*SE* = 2.14, 95% CI = 19.96–28.37 points), and there was a significant change in slope according to the Davies test (*p* = 0.012). The breakpoint of 24.17 NPI points almost perfectly aligns with the onset of the top 10% of the NPI distribution (raw score of 24 corresponds to 90% cumulative frequency). **Figure 3** shows the breakpoint model. The correlations below and above the breakpoint were *r* = -0.09 (*n* = 910, *p* = 0.007, 95% CI = -0.16 to -0.03) and *r* = 0.20 (*n* = 94, *p* = 0.054, 95% CI = 0-0.01 to 0.41). The difference between both was significant according to Steiger’s *z*-test (*z* = -2.66, *p* = 0.004).

## Discussion

This study investigated the relationship between grandiose and vulnerable narcissism (the “two faces” of narcissism; Wink, 1991, p. 590) in a large non-clinical sample. We hypothesized that (1) the common core of grandiose and vulnerable (hypersensitive) narcissism is masked by interindividual differences of intro-/extraversion, a more broad and fundamental personality trait, and that (2) the correlation between grandiose and vulnerable narcissism increases toward the upper end of the narcissism distribution. Latent variable analyses and segmented regression analyses confirmed both of these hypotheses, which shall be discussed in the following.

### Intro-/Extraversion Masks the Common Core of Grandiose and Vulnerable Narcissism

Many previous studies demonstrated that grandiose and vulnerable (hypersensitive) narcissism are essentially unrelated within the range of normal personality variation, but both display substantial correlation with extra- and introversion, respectively (e.g., Miller et al., 2011; see Introduction). This suggests that the fundamental personality trait of intro-/extraversion masks the common core of grandiose and vulnerable narcissism. We investigated this hypothesis by means of latent variable structural equation modeling. Analyses confirmed that the near-zero correlation of *r* = -0.09 rises to 0.53 when controlling for interindividual differences in intro-/extraversion. This means that, if all individuals would display average intro-/extraversion (thus being neither markedly intro-, nor extraverted), they would essentially exhibit increases in vulnerable narcissism alongside grandiose narcissism.

Of course, such a scenario is not realistic, as there actually is substantial variation in intro-/extraversion in the general population. Nonetheless, it may still help to better understand the differentiation of grandiose and vulnerable narcissism in the general population: intro-/extraversion might act as a moderating factor in the development of narcissism. As argued above, intro-/extraversion is a highly genetically determined trait with a clear biological basis (Eysenck, 1967; McCartney et al., 1990; Loehlin, 1992), while narcissism is thought to be more driven by social influences such as parenting style (e.g., Otway and Vignoles, 2006; Brummelmann et al., 2015a,b). It is thus reasonable to think of extra- and introversion as different (biological) fundaments for the possible (later) development of different forms of narcissism, which can be conceived more narrow (specific) personality traits. When exposed to parenting styles and sociocultural factors that convey core narcissistic beliefs (“I am superior”), extraverted and introverted individuals might respond differently, either developing a more extraverted (grandiose; “I am superior and I will let you know it”) or introverted (vulnerable; “Secretly, I know that I am superior”) narcissistic personality.

It has to be noted, though, that behavioral genetic twin studies estimate the heritability of (grandiose) narcissism similar to that of intro-/extraversion (e.g., *a*^2^ = 0.59; Vernon et al., 2008), which may appear to stand at odds with our interpretation at first glance. However, there is substantial *genetic* correlation between grandiose narcissism and extraversion (*r*g = 0.42), which means that “observed correlations […] between the Dark Triad and the Big 5 are largely attributable to the influence of the same genes” (ibid., p. 451). This further strengthens our view that removing intro-/extraversion-variance in narcissism measures unveils the narcissistic core. If this core, in itself, might also depend upon genetic factors has, to our knowledge, not yet been investigated, but would be an intriguing research question. From the considerations presented above, it could be hypothesized that heritability estimates of narcissism measures might be substantially lower when controlling for intro-/extraversion (and, to a lesser extent, also neuroticism).

We put forward the hypothesis that intro-/extraversion acts as a moderating variable in the development of grandiose and vulnerable narcissism. Our data cannot directly test this moderation hypothesis, as it cannot speak to the common social-developmental factor(s) that might promote both narcissistic personality tendencies. However, other research suggests parental overvaluation (Brummelmann et al., 2015a,b) and parental coldness (Otway and Vignoles, 2006) during childhood as possible candidates. Future studies could investigate the moderation hypothesis more directly using childhood recollections of parenting style alongside measures of grandiose and vulnerable narcissism to elucidate the moderating role of intro-/extraversion in the development of narcissism.

Our main hypothesis focused on intro-/extraversion as a control variable in the relationship between grandiose and vulnerable narcissism, but it could also be argued that other Big Five traits are relevant as well. Above all, neuroticism is also frequently reported to correlate negatively with grandiose, but positively with vulnerable narcissism (e.g., Miller et al., 2011, 2015; see also Gore and Widiger, 2016). Thus, we also tested the role of neuroticism (i.e., emotionality from the HEXACO model) as a potential control variable. Neuroticism was also substantially associated with grandiose (*r* = -0.45) and vulnerable (*r* = 0.37) narcissism on a latent level, but did not help to explain the covariance between both as good as intro-/extraversion did (corrected correlations of *r* = 0.21 for neuroticism vs. 0.53 for intro-/extraversion). When both variables were simultaneously considered, neuroticism only mildly increased the residual correlation between grandiose and vulnerable narcissism to 0.63 (as compared to 0.53 for intro-/extraversion alone). This strengthens our view that the results are actually specific to intro-/extraversion (in terms of discriminant validity), and grandiose and vulnerable (hypersensitive) narcissism are better understood in terms of extra- and introverted narcissistic styles rather than emotionally stable and unstable styles. Recently, however, Miller et al. (2017) found that neuroticism was suited better than extraversion to differentiate narcissistic grandiosity and vulnerability. This divergence might be attributed to the different narcissism measures used: while we used the NPI and HSNS, the long-time standard measures in personality research on (subclinical) narcissism, Miller and colleagues used more clinically oriented narcissism measures (amongst them the PNI), thus putting more weight on maladaptive and dysfunctional aspects of narcissism. The different conceptions (subclinical vs. pathological narcissism) of the measures used in both studies are likely to account for the different findings, as neuroticism might be more relevant to the maladaptive aspects associated with pathological narcissism.

The view that different measures of narcissism put weight on different aspects has most recently been expressed in the *Narcissism Spectrum Model* (Krizan and Herlache, 2017). This model assumes that phenotypical differences in the expression of narcissism can be organized on a spectrum ranging from grandiosity to vulnerability; at the core stand entitlement and self-importance. Our data are in very good accordance with this model, as we found that controlling for intro-/extraversion reveals a common core of measures oriented toward narcissistic grandiosity (NPI) and vulnerability (HSNS); as predicted by the model. To this end, it is important to emphasize that hypersensitive narcissistic vulnerability, as measured by the HSNS, appears to be driven more by introversion than neuroticism. This appears perfectly reasonable given that increased sensitivity to environmental stimuli is a constituent feature of introversion (Eysenck, 1967; Kagan, 1994) and underpins the importance of a clear terminology in the light of the manifold measures of narcissism.

### High Grandiose Narcissism Goes along with Vulnerable Aspects

Our second hypothesis concerned the likely non-linear relationship between grandiose and vulnerable narcissism in the general population. We hypothesized that high grandiose narcissism would go along with increased vulnerable aspects, as clinical research indicates that pathological narcissism encompasses both, grandiose and vulnerable aspects (Pincus et al., 2009; Pincus and Lukowitsky, 2010). Segmented regression analysis confirmed this hypothesis, as we observed a significant change in slope at the onset of the top 10% of the NPI distribution. While the correlation below this breakpoint was slightly negative and resembled previous findings (e.g., Ksinan and Vazsonyi, 2016) as well as those from our whole-sample SEM analysis, there was a moderate positive correlation among the upper 10% of the NPI distribution. This implies that in highly narcissistic individuals, grandiose self-states are more likely to be accompanied by vulnerable self-states (Pincus and Lukowitsky, 2010; Gore and Widiger, 2016). These opposing tendencies will presumably lead to experiences of cognitive and affective dissonance, which parallels previous findings showing that high narcissism goes along with discrepancies such as high explicit but low implicit self-esteem (Jordan et al., 2003).

Interestingly, grandiose and vulnerable narcissism are correlated toward the upper end of the distribution regardless of interindividual differences in intro-/extraversion. We hypothesize that this might be the case because narcissistic tendencies, once pronounced enough, might overrule more fundamental personality traits such as intro-/extraversion. That is, as soon as a critical threshold (top 10% of the NPI distribution) is exceeded, individuals who are habitually extraverted might experience episodes of social withdrawal and avoidance more frequently. In statistical terms, adjusting for interindividual differences in intro-/extraversion should have less effect on the correlation between grandiose and vulnerable narcissism beyond the onset of the upper 10%. However, we cannot directly test this supposition as measures of intro-/extraversion were only available in a smaller subsample in this study (data on intro-/extraversion is available for less than 50 individuals beyond the NPI-breakpoint).

### Limitations and Prospects

As noted above, our analysis of intro-/extraversion as a control variable in the relationship between grandiose and vulnerable (hypersensitive) narcissism can provide first insights into the complex interplay of these personality traits, but future studies should directly address the issue of intro-/extraversion as a moderating variable in the development of narcissism. This could be accomplished, for instance, by testing interactive effects of parenting style – a presumed developmental factor of narcissism (Otway and Vignoles, 2006; Brummelmann et al., 2015a,b) – and intro-/extraversion onto the two narcissism constructs.

From a critical perspective, one could argue that controlling for interindividual differences in intro-/extraversion might distort the narcissism variables in an unrealistic fashion (i.e., grandiose narcissism is no longer grandiose without extraversion), which would render the residual correlation meaningless. As argued above, however, controlling for differences in intro-/extraversion can also be viewed as a removal of variance that cannot only be attributed to the specific narcissism measures, but also to the more fundamental personality trait of intro-/extraversion (in terms of general self- or other-orientation). Importantly, we do *not* consider the statistically corrected variables more “pure” indicators of narcissism. Instead, the statistical correction used here should be considered a thought experiment that might be useful for further theory building: what would remain, if we would subtract extraversion from narcissistic grandiosity and introversion from vulnerability? It is the narcissistic core, distilled from broader personality traits, and this core is the same in both cases.

Importantly, the interpretation of the results reported here is restricted to the specific personality measures used: we used the NPI and the HSNS, which can be conceived the long-time standard measures of grandiose and vulnerable (hypersensitive) narcissism. Our results show that these are most clearly differentiated by means of intro-/extraversion. However, using other measures (and thus conceptions) of narcissism might lead to different findings. For instance, grandiose and vulnerable aspects are intrinsically correlated in the PNI (Pincus et al., 2009), and their differentiation might depend more upon neuroticism (Miller et al., 2017). Another prominent model of (grandiose) narcissism distinguishes narcissistic admiration from rivalry (Back et al., 2013). Though rivalry encompasses some features of narcissistic vulnerability, it appears to tap more into the antagonistic aspects (Krizan and Herlache, 2017). Future studies could use a multidimensional approach to discern the manifold conceptions of narcissism.

It also has to be noted that the correlation of *r* = 0.20 reported here for the subsample of the top 10% in the NPI distribution is of comparable magnitude to full-scale correlations occasionally obtained in other studies (e.g., Given-Wilson et al., 2011; Zajenowski et al., 2016). However, the majority of previous studies reported zero correlation, and, to our knowledge, this is the first study to investigate a non-linear association between grandiose and vulnerable narcissism. If the method would be applied to previous datasets, it might turn out that the correlation increases toward the upper end of the distribution as well (and might be even higher in datasets where the average correlation is higher). Also, as Gore and Widiger (2016) emphasize, grandiosity and vulnerability may also be understood in terms of states, not only traits. These states can fluctuate in the way that grandiose narcissists experience episodes of increased vulnerability and social withdrawal (ibid.). From this point of view, one would not expect a very high but rather a moderate correlation between the two trait measures, as the individual’s self-concept may still be dominated by grandiosity and vulnerable aspect might be reported with greater reluctance.

Lastly, many previous studies found substantial sex differences in the association between narcissism and emotion regulation, suggesting that primarily men display emotional deficits associated with narcissism (Morf and Rhodenwalt, 2001; Edelstein et al., 2010; Reinhard et al., 2012; Jauk et al., 2016a, 2017). Thus, it could be hypothesized that the association between grandiose and vulnerable aspects toward the upper end of the distribution is stronger in men. The sample investigated in this study, although large, did not allow for a powerful test of this hypothesis, but future studies could address this question.

## Conclusion

From this study, it can be concluded that grandiose and vulnerable (hypersensitive) narcissism are not distinct traits, but rather different manifestations of the same phenomenon, which aligns well with clinical theories of narcissism. Whether individuals actually display grandiose or vulnerable narcissism at a subclinical level depends on intro-/extraversion, a more basal personality trait. However, at high levels of narcissism (upper 10% of the NPI distribution), narcissistic grandiosity is more likely to be accompanied by narcissistic vulnerability, which could indicate a possible transition topathological narcissism.

## Author Contributions

EJ and EW developed the study concept. EJ, EW, KL, MB, and AN contributed to the study design. Testing and data collection were performed by EJ, EW, and KL. EJ, EW, and KL performed the data analysis and interpretation under the supervision of AN. EJ, EW, and KL drafted the manuscript, MB and AN provided critical revisions. All authors approved the final version of the manuscript for submission.

## Conflict of Interest Statement

The authors declare that the research was conducted in the absence of any commercial or financial relationships that could be construed as a potential conflict of interest.

## Footnotes

1 We use terms such as “personality types,” “grandiose narcissists,” or “vulnerable narcissists” for illustrative purposes though the underlying traits are continuous.

2 The NPI captures maladaptive aspects of narcissism (Ackerman et al., 2011) and displays high concurrent validity with expert ratings of NPD (Miller et al., 2014a). More generally, it is also known from other fields of personality research that extreme scores are likely to reflect psychopathological traits (e.g., extreme conscientiousness; Carter et al., 2016).

3 Error correlations were specified according to modification indices between the facets *social self-esteem*^∗^*social boldness*, *social self-esteem*^∗^*sociability*, *social boldness*^∗^*sociability*, *sociability*^∗^*liveliness*. Correlations were in the range of -0.33 (*social self-esteem*^∗^*social boldness*) to 0.28 (*sociability*^∗^*liveliness*). Omitting the error correlations would have worsened model fit significantly [Satorra–Bentler – scaled difference test: Δχ^2^(12) = 72.19, *p* < 0.001], but did not affect the coefficients of interest. Thus, we included error correlations in the model.

4 Complemental analyses with different initial guess parameters yielded the same results.

## References

[B1] Ackerman R. A. Witt E. A. Donnellan M. B. Trzesniewski K. H. Robins R. W. Kashy D. A. (2011). What does the narcissistic personality inventory really measure? *Assessment* 18 67–87. 10.1177/1073191110382845 20876550

[B2] Anderson J. C. Gerbing D. W. (1988). Structural equation modeling in practice: a review and recommended two-step approach. *Psychol. Bull.* 103 411–423. 10.1037/0033-2909.103.3.411

[B3] Ashton M. C. Lee K. (2009). The HEXACO-60: a short measure of the major dimensions of personality. *J. Pers. Assess.* 91 340–345. 10.1080/00223890902935878 20017063

[B4] Back M. D. Küfner A. C. P. Dufner M. Gerlach T. M. Rauthmann J. F. Denissen J. J. A. (2013). Narcissistic admiration and rivalry: disentangling the bright and dark sides of narcissism. *J. Pers. Soc. Psychol.* 105 1013–1037. 10.1037/a0034431 24128186

[B5] Back M. D. Schmukle S. C. Egloff B. (2010). Why are narcissists so charming at first sight? Decoding the narcissism–popularity link at zero acquaintance. *J. Pers. Soc. Psychol.* 98 132–145. 10.1037/a0016338 20053038

[B6] Beauducel A. Wittmann W. W. (2005). Simulation study on fit indexes in CFA based on data with slightly distorted simple structure. *Struct. Equ. Modeling* 12 41–75. 10.1207/s15328007sem1201_3

[B7] Benedek M. Jauk E. Sommer M. Arendasy M. Neubauer A. C. (2014). Intelligence, creativity, and cognitive control: the common and differential involvement of executive functions in intelligence and creativity. *Intelligence* 46 73–83. 10.1016/j.intell.2014.05.007 25278640 PMC4175011

[B8] Brown A. A. Freis S. D. Carroll P. J. Arkin R. M. (2016). Perceived agency mediates the link between the narcissistic subtypes and self-esteem. *Pers. Individ. Dif.* 90 124–129. 10.1016/j.paid.2015.10.055

[B9] Brummelmann E. Thomaes S. Nelemans S. A. Orobio de Castro B. Bushman B. J. (2015a). My child is god’s gift to humanity: development and validation of the parental overvaluation scale (POS). *J. Pers. Soc. Psychol.* 108 665–679. 10.1037/pspp0000012 25365035

[B10] Brummelmann E. Thomaes S. Nelemans S. A. Orobio de Castro B. Overbeek G. Bushman B. J. (2015b). Origins of narcissism in children. *Proc. Natl. Acad. Sci. U.S.A.* 112 3659–3662. 10.1073/pnas.1420870112 25775577 PMC4378434

[B11] Buss D. M. Chiodo L. M. (1991). Narcissistic acts in everyday life. *J. Pers.* 59 179–215. 10.1111/j.1467-6494.1991.tb00773.x 1880699

[B12] Carter N. T. Guan L. Maples J. L. Williamson R. L. Miller J. D. (2016). The downsides of extreme conscientiousness for psychological well-being: the role of obsessive compulsive tendencies. *J. Pers.* 84 510–522. 10.1111/jopy.12177 25858019

[B13] Corry N. Merritt R. D. Mrug S. Pamp B. (2008). The factor structure of the narcissistic personality inventory. *J. Pers. Assess.* 90 593–600. 10.1080/00223890802388590 18925501

[B14] Davies R. B. (1987). Hypothesis testing when a nuisance parameter is present only under the alternative. *Biometrika* 74 33–43.

[B15] Dufner M. Rauthmann J. F. Czarna A. Z. Denissen J. J. A. (2013). Are narcissists sexy? Zeroing in on the effect of narcissism on short-term mate appeal. *Pers. Soc. Psychol. Bull.* 39 870–882. 10.1177/0146167213483580 23554177

[B16] Edelstein R. S. Yim I. S. Quas J. A. (2010). Narcissism predicts heightened cortisol reactivity to a psychosocial stressor in men. *J. Res. Pers.* 44 565–572. 10.1016/j.jrp.2010.06.008 21076653 PMC2976540

[B17] Emmons R. A. (1984). Factor analysis and construct validity of the narcissistic personality inventory. *J. Pers. Assess.* 48 291–300. 10.1207/s15327752jpa4803_11 16367528

[B18] Eysenck H. J. (1967). *The Biological Basis of Personality.* New Brunswick: Transaction Publishers.

[B19] Fossati A. Borroni S. Grazioli F. Dornetti L. Marcassoli I. Maffei C. (2009). Tracking the hypersensitive dimension in narcissism: reliability and validity of the hypersensitive narcissism scale. *Pers. Ment. Health* 3 235–247. 10.1002/pmh.92

[B20] Given-Wilson Z. McIlwain D. Warburton W. (2011). Meta-cognitive and interpersonal difficulties in overt and covert narcissism. *Pers. Individ. Dif.* 50 1000–1005. 10.1016/j.paid.2011.01.014

[B21] Gore W. L. Widiger T. A. (2016). Fluctuation between grandiose and vulnerable narcissism. *Pers. Disord.* 7 363–371. 10.1037/per0000181 26986960

[B22] Hart W. Adams J. Burton K. A. Tortoriello G. K. (2017). Narcissism and self-presentation: Profiling grandiose and vulnerable narcissists’ self-presentation tactic use. *Pers. Individ. Dif.* 104 48–57. 10.1016/j.paid.2016.06.062

[B23] Haybach G. Küchenhoff H. (1997). *Testing for a breakpoint in Two-Phase Linear and Logistic Regression Models.* Berlin: Sonderforschungsbereich.

[B24] Hendin H. M. Cheek J. M. (1997). Assessing hypersensitive narcissism: a reexamination of Murray’s Narcism scale. *J. Res. Pers.* 31 588–599. 10.1006/jrpe.1997.2204

[B25] Hu L. T. Bentler P. M. (1998). Fit indices in covariance structure modeling: sensitivity to underparameterized model misspecification. *Psychol. Methods* 3 424–453. 10.1037/1082-989X.3.4.424

[B26] Hu L. T. Bentler P. M. (1999). Cutoff criteria for fit indexes in covariance structure analysis: conventional criteria versus new alternatives. *Struct. Equ. Modeling* 6 1–55. 10.1080/10705519909540118

[B27] Jauk E. Benedek M. Dunst B. Neubauer A. C. (2013). The relationship between intelligence and creativity: new support for the threshold hypothesis by means of empirical breakpoint detection. *Intelligence* 41 212–221. 10.1016/j.intell.2013.03.003 23825884 PMC3682183

[B28] Jauk E. Benedek M. Koschutnig K. Kedia G. Neubauer A. C. (2017). Self-viewing is associated with negative affect rather than reward in highly narcissistic men: an fMRI study. *Sci. Rep.* 7 5804 10.1038/s41598-017-03935-y PMC5517462 28724894

[B29] Jauk E. Freudenthaler H. H. Neubauer A. C. (2016a). The Dark Triad and trait versus ability emotional intelligence: emotional darkness differs between women and men. *J. Individ. Differ.* 37 112–118. 10.1027/1614-0001/a000195

[B30] Jauk E. Neubauer A. C. Mairunteregger T. Pemp S. Sieber K. P. Rauthmann J. F. (2016b). How alluring are dark personalities? The Dark Triad and attractiveness in speed dating. *Eur. J. Pers.* 30 125–138. 10.1002/per.2040

[B31] Jordan C. H. Spencer S. J. Zanna M. P. Hoshino-Browne E. Correll J. (2003). Secure and defensive high self-esteem. *J. Pers. Soc. Psychol.* 85 969–978. 10.1037/0022-3514.85.5.969 14599258

[B32] Kagan J. (1994). *Galen’s Prophecy.* New York, NY: Basic Books.

[B33] Karwowski M. Dul J. Gralewski J. Jauk E. Jankowska D. M. Gajda A. (2016). Is creativity without intelligence possible? A necessary condition analysis. *Intelligence* 57 105–117. 10.1016/j.intell.2016.04.006

[B34] Kernberg O. F. (1975). *Borderline Conditions and Pathological Narcissism.* New York, NY: Aronson.

[B35] Kohut H. (1977). *The Restoration of the Self.* Madison: International Universities Press.

[B36] Krizan Z. Herlache A. D. (2017). The narcissism spectrum model: a synthetic view of narcissistic personality. *Pers. Soc. Psychol. Rev.* 10.1177/1088868316685018 [Epub ahead of print]. 28132598

[B37] Ksinan A. J. Vazsonyi A. T. (2016). Narcissism, internet, and social relations: a study of two tales. *Pers. Individ. Dif.* 94 118–123. 10.1016/j.paid.2016.01.016

[B38] Kubarych T. S. Deary I. J. Austin E. J. (2004). The narcissistic personality inventory: factor structure in a non-clinical sample. *Pers. Individ. Dif.* 36 857–872. 10.1016/S0191-8869(03)00158-2

[B39] Lee K. Ashton M. C. (2005). Psychopathy, machiavellianism, and narcissism in the five-factor model and the HEXACO model of personality structure. *Pers. Individ. Dif.* 38 1571–1582. 10.1016/j.paid.2004.09.016

[B40] Loehlin J. C. (1992). *Genes and Environment in Personality Development.* Newbury Park: Sage.

[B41] McCartney K. Harris M. J. Bernieri F. (1990). Growing up and growing apart: a developmental meta-analysis of twin studies. *Psychol. Bull.* 107 226–237. 10.1037/0033-2909.107.2.226 2138795

[B42] Miller J. D. Hoffman B. J. Gaughan E. T. Gentile B. Maples J. Campbell W. K. (2011). Grandiose and vulnerable narcissism: a nomological network analysis. *J. Pers.* 79 1013–1042. 10.1111/j.1467-6494.2010.00711.x 21204843

[B43] Miller J. D. Lynam D. R. Hyatt C. S. Campbell W. K. (2017). Controversies in narcissism. *Annu. Rev. Clin. Psychol.* 13 291–315. 10.1146/annurev-clinpsy-032816-045244 28301765

[B44] Miller J. D. Lynam D. R. McCain J. L. Few L. R. Crego C. Widiger T. A. (2015). Thinking structurally about narcissism: an examination of the five-factor narcissism inventory and its components. *J. Pers. Disord.* 30 1–18. 10.1521/pedi_2015_29_177 25710734

[B45] Miller J. D. McCain J. Lynam D. R. Few L. R. Gentile B. MacKillop J. (2014a). A comparison of the criterion validity of popular measures of narcissism and narcissistic personality disorder via the use of expert ratings. *Psychol. Assess.* 26 958–969. 10.1037/a0036613 24773036

[B46] Miller J. D. Price J. Gentile B. Lynam D. R. Campbell W. K. (2012). Grandiose and vulnerable narcissism from the perspective of the interpersonal circumplex. *Pers. Individ. Dif.* 53 507–512. 10.1080/00223891.2012.742903

[B47] Miller J. D. Widiger T. A. Campbell W. K. (2014b). Vulnerable narcissism: commentary for the special series “narcissistic personality disorder—new perspectives on diagnosis and treatment”. *Pers. Disord.* 5 450–451. 10.1037/per0000083 25314236

[B48] Morf C. C. Rhodenwalt F. (2001). Unraveling the paradoxes of narcissism: a dynamic self-regulatory processing model. *Psychol. Inq.* 14 177–196. 10.1207/S15327965PLI1204_1

[B49] Mueggo V. M. (2008). Segmented: an R package to fit regression models with broken-line relationships. *R News* 8 20–25.

[B50] Ng H. K. S. Tam K.-P. Shu T.-M. (2011). The money attitude of covert and overt narcissists. *Pers. Individ. Dif.* 51 160–165. 10.1016/j.paid.2011.03.036

[B51] Otway L. Vignoles V. L. (2006). Narcissism and childhood recollections: a quantitative test of psychoanalytic predictions. *Pers. Soc. Psychol. Bull.* 32 104–116. 10.1177/0146167205279907 16317192

[B52] Paulhus D. L. (2001). Normal narcissism: two minimalist accounts. *Psychol. Inq.* 12 228–230.

[B53] Paulhus D. L. Williams K. M. (2002). The dark triad of personality: narcissism, machiavellianism, and psychopathy. *J. Res. Pers.* 36 556–563. 10.1016/S0092-6566(02)00505-6

[B54] Pincus A. L. Ansell E. B. Pimentel C. A. Cain N. M. Wright A. G. C. Levy K. N. (2009). Initial construction and validation of the pathological narcissism inventory. *Psychol. Assess.* 21 365–379. 10.1037/a0016530 19719348

[B55] Pincus A. L. Cain N. M. Wright A. G. C. (2014). Narcissistic grandiosity and narcissistic vulnerability in psychotherapy. *Pers. Disord.* 5 439–443. 10.1037/per0000031 24446581

[B56] Pincus A. L. Lukowitsky M. R. (2010). Pathological narcissism and narcissistic personality disorder. *Annu. Rev. Clin. Psychol.* 6 421–446. 10.1146/annurev.clinpsy.121208.131215 20001728

[B57] Raskin R. Hall C. S. (1979). A Narcissistic Personality Inventory. *Psychological Reports* 45 590 10.2466/pr0.1979.45.2.590 538183

[B58] Raskin R. Hall C. S. (1981). The Narcissistic Personality Inventory: Alternative form reliability and further evidence of construct validity. *J. Pers. Assess.* 45 159–162. 10.1207/s15327752jpa4502_10 16370732

[B59] Reinhard D. A. Konrath S. Lopez W. D. Cameron H. G. (2012). Expensive egos: narcissistic males have higher cortisol. *PLOS ONE* 7:e30858 10.1371/journal.pone.0030858 PMC3264640 22292062

[B60] Roberts C. R. D. Huprich S. K. (2012). Categorical and dimensional models of pathological narcissism: the case of Mr. Jameson. *J. Clin. Psychol.* 68 898–907. 10.1002/jclp.21894 22730014

[B61] Roche M. J. Pincus A. L. Lukowitsky M. R. Ménard K. S. Conroy D. E. (2013). An integrative approach to the assessment of narcissism. *J. Pers. Assess.* 95 237–248. 10.1080/00223891.2013.770400 23451709

[B62] Schütz A. Marcus B. Sellin I. (2004). Die messung von narzissmus als persönlichkeitskonstrukt: psychometrische eigenschaften einer lang- und einer kurzform des deutschen NPI (Narcissistic Personality Inventory). *Diagnostica* 50 202–218. 10.1026/0012-1924.50.4.202

[B63] Thomaes S. Brummelmann E. Reijntjes A. Bushman B. J. (2013). When narcissus was a boy: origins, nature, and consequences of childhood narcissism. *Child Dev. Perspect.* 7 22–26. 10.1111/cdep.12009

[B64] Twenge J. M. Campbell W. K. (2009). *The Narcissism Epidemic: Living in the Age of Entitlement.* New York, NY: Free Press.

[B65] Twenge J. M. Foster J. D. (2010). Birth cohort increases in narcissistic personality traits among American college students, 1982-2009. *Soc. Psychol. Pers. Sci.* 1 99–106. 10.1177/1948550609355719

[B66] Ulm K. (1991). A statistical model for assessing a threshold in epidemiology. *Stat. Med.* 10 341–349. 10.1002/sim.4780100306 2028118

[B67] Vernon P. A. Villani V. C. Vickers L. C. Harris J. A. (2008). A behavioral genetic investigation of the Dark Triad and the big 5. *Pers. Individ. Dif.* 44 445–452. 10.1016/j.paid.2007.09.007

[B68] Wink P. (1991). Two faces of narcissism. *J. Pers. Soc. Psychol.* 61 590–597. 10.1037/0022-3514.61.4.590 1960651

[B69] Zajenowski M. Witowska J. Maciantowicz O. Malesza M. (2016). Vulnerable past, grandiose present: the relationship between vulnerable and grandiose narcissism, time perspective and personality. *Pers. Individ. Dif.* 98 102–106. 10.1016/j.paid.2016.03.092

